# Supplementary material for: Predicting primate tongue morphology based on geometrical skull matching. A first step towards an application on fossil hominins
Source: PLoS Comput Biol. 2024 Jan 22;20(1):e1011808. doi: 10.1371/journal.pcbi.1011808 (PMC10833839; doi:10.1371/journal.pcbi.1011808)
Supplement: Attachment — Submitted filename: Answers_to_reviewers.pdf [file pcbi.1011808.s001.pdf]

Paper No.: PCOMPBIOL-D-23-01170

Title: *Predicting primate tongue morphology based on geometrical skull matching. A first step towards an application on fossil hominins*

We thank the editor and both reviewers for their valuable comments and suggestions. Our answers are written in blue font. We have attached the revised manuscript at the end of this letter with changes highlighted also in blue font. Please bear in mind that many of the changes correspond to improvements of the text in general. For this reason, in this document, we explicitly mention the relevant sections/paragraphs of the text that were revised according to the reviewers' remarks, when necessary.

Reviewer #1:

The authors present a compelling case for the feasibility of predicting unknown tongue morphology by aligning 3D CT images of head/neck bone structures (serving as a substitute for fossil records) with human CT images. This research lays the groundwork for reconstructing the soft tissue of the upper vocal tract from hominin fossils, opening the door to unraveling the evolutionary biomechanics of language production. This methodology holds promise and could be of significant importance for the field of paleoanthropology. However, I have a few minor concerns regarding the approach:

1. The study employs a baboon as a surrogate for hominin variation, yet the baboon's morphology diverges considerably from that of apes, which are closer relatives to extinct hominins. For instance, the elongated muzzle of the baboon, not present in hominins, could account for much of the observed transformation. How does the performance of the method correlate with these morphological differences? One suggestion would be to assess the method's efficacy using simulated hominin-like images, generated by warping baboon or human images.

Besides the multi-resolution registration approach, we account for large deformation by means of minimizing the distance between paired anatomical landmarks (see Sec. 2.2.3.2). For all our registration results, the final distance between these landmarks is smaller than 1 mm in average. Since some landmarks were placed close to the tip of the muzzle (see landmarks 1 and 2 in Fig. 1), a low distance for these landmarks after registration is an indication of a good estimation of the large postero-anterior deformation induced by the muzzle. This is confirmed in Figs. 5 and 6, where it can be clearly observed that our tongue predictions fill up the oral cavity in a plausible manner.

As the reviewer mentions, the differences between baboon and modern human morphologies are considerably larger than those between modern and extinct human morphologies. Therefore, we argue that the capacity of our registration method to properly account for these large morphological differences is extremely promising in terms of the method's ability to account for smaller differences in the context of the prediction of fossil hominins' tongues. To illustrate this point, we have predicted the tongue morphology of a living patient with mandibular retrognathia, for which qualitative results are depicted in Fig. A. For this new estimation, we used the exact optimal registration parameters resulting from the uncertainty quantification procedure for the *bones-based* registration problem ($\lambda_1 = 25.38 \text{ mm}, \lambda_2 = 844.35$).

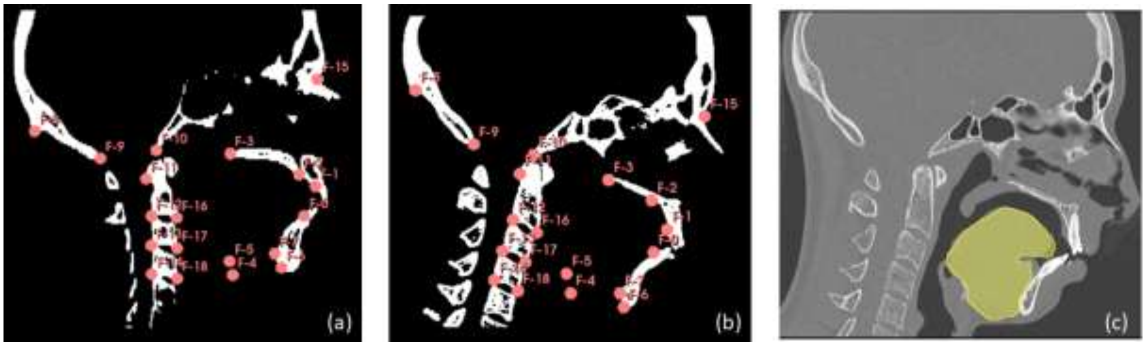

Figure A: Estimated tongue for a new modern human subject using bone-based registration. (a) Mid-sagittal plane of the reference modern human CT with only bone information. (b) Mid-sagittal plane of the new modern human CT with only bone information. (c) Resulting outline of FE tongue superimposed on mid-sagittal plane of the other modern human original CT.

We are conscious that the publication of the qualitative results presented in Fig. A could greatly support the results already presented in the text. Unfortunately, the CT images from the patient are part of a pilot study in which the patient was volunteer but which is not covered by any ethical agreement. Consequently, these results cannot be published, and are used here for illustration purposes only.

Finally, given the morphological proximity between modern and extinct humans, we expect the proposed registration approach to perform well in this context too. We illustrate this in Fig. B, below.

2. Could you provide more details on how the error size compares with the range of natural variability within the species? Understanding this relationship would help contextualize the study's results.

At present, to our knowledge, quantitative studies of morphological variability of the oral cavity and associated structures is lacking for baboons.

However, several studies have shown that differences of up to several millimeters may exist between adult individuals at all stages of human's evolution (see for example [Pearson1924]). Experimental work on this variability in modern humans is currently underway, but will require considerable work.

3. In studies concerning the evolution of language, the position of the hyoid bone is another critical factor to consider. How accurate is the prediction of the hyoid bone's position when using the bone-based approach? This additional metric could further validate the methodology.

The proposed registration approach uses paired anatomical landmarks at the hyoïd bone, for both the *soft-tissue-based* and *bones-based* registration problems. Therefore, in the present study, the predicted position for the hyoïd bone is highly accurate with respect to these landmarks. This, however, supposes that the hyoïd bone's position is known, which may seem dubious since for extinct species it is not. There is no consensus among paleontologists regarding its estimated position neither [10, 17]. However, some guidelines have been proposed [2, 6] and we plan to use them as reference in the short-term. More importantly, the quantitative study of the impact of variations in the estimation of the hyoid bone position, based on bony structures alone, is part of our research project based on the use of predicted tongue models of fossil humans on the capacity to articulate distinctive sounds, which are at the basis of the emergence of spoken language.

Changes:

– A comment has been added at the end of the discussion regarding future work. We found the before-last paragraph of the discussion to answer well to this question.

Overall, the study is intriguing, but addressing these points could strengthen the implications of the work.

We thank the reviewer for the insightful comments, and we hope to have answered to all the raised concerns.

Reviewer #2:

The study aims to predict primate tongue morphology using geometric skull matching, with a potential application to fossil hominins. Authors propose a bone-based nonlinear image registration approach since for fossil hominins, only bones of the skull and mandible are preserved. The results are satisfactory when compared with the “golden truth” of the baboon tone segmented from the CT image. The study is well structured and well written, and it’s a pleasure for me to read. Especially, the authors’ long-term goal of the project is intriguing and very interesting, aiming for a quantitative evaluation of the biological characteristics of fossil hominins with the capacity of articulated speech. However, there are some major concerns about the design of the study.

We thank the reviewer for such a positive appreciation. We provide below detailed answers to all the reviewer’s remarks.

1. A major concern is the validity of the model applied to fossil hominins. While the study successfully predicts baboon tongue morphology using the chosen method, it's unclear whether this approach is valid for predicting tongues of fossil hominins. However, this assumption may oversimplify the complex relationship between skeletal and soft tissue structures, and the applicability of this model to extinct species with different craniofacial and vocal tract configurations requires further justification. Though the authors have attempted to motivate their choices (lines 110-116). I still find it difficult to be convinced. Especially authors might want to revise the following sentence: *“we are confident that the proposed bone-based registration approach is powerful enough to make possible the prediction of plausible biomechanical tongue models of fossil hominins…”*

We understand the reviewer’s skepticism and we agree that further validation will be needed when applying the proposed registration approach to fossil specimens. However, few morphological differences have been reported in the literature among mammal tongues [Iwasaki2019], which leads us to believe that such estimation is possible.

In Fig. B, we present a preliminary result for the estimation of a FE tongue mesh of a neanderthal specimen (“La Férassie 1”). Please note that a symmetrization process was carried out for the fossil data, so that our proposed approach is directly applicable. A new research article is underway to report this, and other results for fossil data, as well as a detailed description of the process. We consider that the publication of the first biomechanical model of a Neanderthalian deserves a specific paper. We don’t want to integrate these results in the current manuscript, which aims is to validate the registration method, that will be used for fossil hominins (see also our response to comment 6 below).

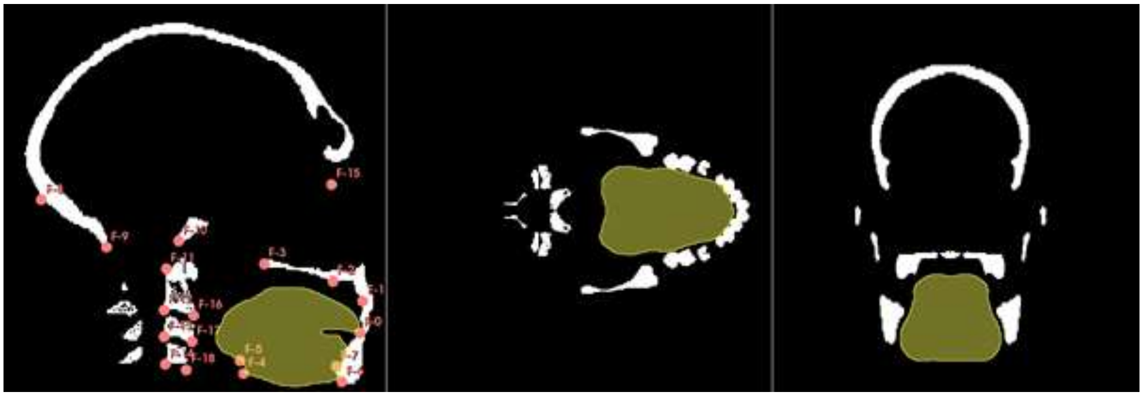

Figure B: Estimated tongue for a symmetrized version of "La Ferrassie 1" fossil specimen. (a) Mid-sagittal plane of the fossil specimen with anatomical landmarks. (b) Axial plane at 9.8 mm above the infradental landmark (F-0). (c) Coronal plane at 37.3 mm behind the nasion landmark (F-15).

Changes:

- The introduction now mentions more explicitly our hypotheses for the transferability from of previous work [3] to our fossil human context.
- The final paragraph of the discussion has been also revised to emphasize the importance of a sensibility analysis in the future.

2. Relating to the above is the choice of image registration method. One of the backup reasons for choosing B-spline registration is based on ref [3]. However, notice the difference of purpose of that study is different from the current one: ref [3] focuses on subject-specific FE mesh generation morphed from an atlas mesh, also between humans. Thus authors might want to revise the following sentences to acknowledge this: *This methodology is inspired from previous work [3] and consists in morphing a human tongue mesh, which was build from medical head and neck images of a reference living human, onto the anatomy of a target subject, using non rigid registration techniques. Bijar and colleagues [3] have validated their approach starting with the prediction of the tongue of another living human.*

We acknowledge that the justification for our methodological choice can be enriched. Before introducing the changes to the text, we note that the answer to this question and that to question 4 are strongly related. We therefore choose to reply to these two questions in a unified manner below.

4. The choice of image registration approach is essential to this task. Authors should consider more registration methods besides the current B-Spline, e.g. Diffeomorphic demons, or other similar. especially one reason for this choice is based on ref [3], while mentioned above, that study had a different focus.

Deformable image registration has been an active research field for decades, and many methods have been proposed for applications with varying degrees of complexity [Sotiras2013]. The choice of one method over the other typically depends on the application at hand, although one can also argue that several methods could lead to similar results. We recall that the purpose of our work is by no means to provide an extensive comparison of deformable image registration methods for our application (which would be practically impossible), but rather to propose a feasible solution to our problem while providing a measure of its uncertainty. In that sense, the choice of a free-form deformation model with cubic B-Splines interpolation is pragmatic. Not only it has been established by previous works that this approach performs generally well for large deformation registration problems [Rueckert2006, Haghighi2018], but it is also fast and easy to parametrize, which facilitates estimating its uncertainty. Furthermore, Bijar and colleagues [3] have demonstrated its feasibility in a context similar to ours: the

estimation of a *new subject*'s tongue via morphing the tongue of a reference human, which is the argument that we put forward in the text. In our context, this *new subject* is a fossil hominin, for which we expect the proposed methodology to perform well, given the morphological proximity of skeletal and soft tissue structures in the oral cavity. However, considering the lack of validation for the fossil hominin, we have chosen to demonstrate the feasibility of our approach in a more challenging setting where the *new subject* is a Baboon.

Nonetheless, there are further, less pragmatic reasons to support our methodological choice for deformable image registration. We recall that transformation models may be classified into nonparametric (all Demons based methods) and parametric (radial basis functions, thin plate splines, free-form deformation with B-Splines, etc.). The main difference between the two being their richness of description: a displacement per voxel for the former as opposed to a reduced set of parameters for the latter. Given the ill-posedness of deformable image registration in general, the larger the number of parameters to estimate the more likely it is for the optimization algorithm to fall into local minima [Sotiras2013]. Although researchers have successfully used nonparametric approaches with the help of regularization techniques, the images to be registered are typically richer in intensity information than those available for our application. A parametric transformation model seemed more appropriate, since by construction, it provides local correlation of displacements, implicit smoothness regularization, and its fewer degrees of freedom facilitate convergence during optimization. As for the choice of the parametric model, free-form deformations with B-Splines are, to our knowledge, the most performing choice thanks to their local support and flexible parametrization, which is probably the reason for their popularity in the medical image registration community [Sotiras2013, Haghighi2018].

Finally, considering our objectives, our reported results and the current length of the text, we believe that a comparison to other deformable image registration methods is out of scope. We therefore tried to better convey the above-mentioned reasoning in the text, by including the following modifications:

Changes:

- Changes in the first part of the abstract whose original formulation could let think that we evaluated two registration methods, whereas our goal is in fact to assess variations in the predicted baboon tongue provided by our proposed method depending on whether soft tissue are taken into account or not, as will be the case for the estimation of fossil humans' tongues.
- The introduction now mentions the reasons why we think non-rigid registration using FFD with B-Splines interpolation should work well.
- Modified section 2.2.3.1 on the choice of transformation model.

3. Another major comment: Given that the study aims to predict morphological and geometrical aspects, the inclusion of finite element meshing may complicate the problem unnecessarily. It might be more effective to exclude the finite element meshing part to focus solely on geometry/morphology. Especially, since meshing a tongue to FE mesh would be easy using e.g. Hypermesh as authors are familiar with especially if authors choose to mesh with tetrahedral elements.

The construction of a high-quality finite element mesh of the tongue accounting for refined internal structures (e.g. 15 oriented muscles components) could have been possible. However, it was important for us to preserve the internal structure of the reference mesh, in which we have carefully specified the anatomical implementation of the tongue muscles, associating elements with muscles and specifying in each element the main direction of muscle forces. This muscle implementation is crucial for further simulations of tongue movements, and of the assessment of tongue mobility and stability in the context of the production of distinctive sounds that could be at the basis of the emergence of

spoken language. In addition, as mentioned above, the influence of crucial parameters of the registration method, such as the position of the hyoïd bone, will require the generation of several models for each fossil hominin. High-quality finite element meshing is very time consuming, and this approach could then be a handicap in our project to systematically evaluate the influence of these parameters in the registration process. .

Changes:

- Section 2.1.2 now includes a brief comment on internal muscle meshing.

5. Related to the above, it would be valuable to explore how different registration methods contribute to uncertainties, supplementing the current uncertainty quantification of parameters.

We believe, as mentioned above, that the inclusion of other registration approaches would not significantly improve our present work. As described in the text, the uncertainty quantification is a time consuming and computationally expensive procedure. In the current work, we only treated two uncertain parameters for two deformable image registration strategies. The inclusion of other registration approaches would significantly increase the number of parameters, leading a problem not only neighboring the untreatable, but also very difficult to present comprehensively in single publication. We emphasize again that this was by no means the objective of our work.

6. Consider adding a fossil hominin skull bone to the study to complete the story and demonstrate the result of a predicted tongue for a fossil hominin.

In Fig. B, we demonstrate the feasibility of the proposed registration approach to predict the tongue of a neanderthal specimen. We deliberately exclude this result from the paper as part of our publication strategy. Indeed, we would like the current article to serve as a methodological basis for later publications dealing specifically with fossil hominins and FE simulations, which will explore research questions from the field of paleoanthropology, and will therefore not be tailored for the same community. It is precisely for this reason that we chose the subtitle "A first step towards an application on fossil hominins".

7. If the authors chose to focus on the geometry aspect, and due to the motivation in lines 110-115, would it benefit to morph the baboon skull/tongue directly to a fossil hominin instead of morphing from a human?

To date, considerable time has been devoted to create a reference model of the human tongue, both in terms of topology and anatomical structures [Calka2023]. The suggested evaluation would require generating such a model for the Baboon, which implies similar upstream work only for this evaluation. We do not believe such evaluation is superior or preferable than that presented in the paper, with the disadvantage of requiring a detailed FE mesh exploitable only for this work, as opposed to our proposed evaluation.

8. Ethical approval statement is lacking, especially since this study involves CT scans of a human and baboon.

The CT images were collected in 2001 from one of our colleagues, who volunteered to be a subject for this study. At the time of the recording no ethical approval was required in France for this kind of data recording, from the moment the subject was volunteer and the physicians agreed to do the recording. These data have already been published in two publications in peer reviewed journals: [Buchaillard2007, Buchaillard2009]

The CT image of the baboon was acquired with approval of the local ethical committee (no. F1308707**)**.

Specific comments

1. Figure 2 seems to show the CT image of the human misses the upper part. Please clarify if this is just an illustration problem or indeed so.

This has been clarified in Sec. 2.1.1.

2. Figure 5(d) seems the predicted tongue covers the void (throat region?) please clarify.

The imperfections in the lowest posterior region of the estimated tongue shape (Fig. 5(d)) are likely due to the orientation of the baboon's head, looking upwards, which generates in this region of the throat a vocal-tract bending that is clearly different from the one in our reference subject. Consequently, the landmarks in the cervical vertebrae for the baboon subject are relatively farther away from the tongue than those for the human reference subject. Since a perfect match of the two morphologies may not be possible without introducing large mesh distortion, the proposed geometrical deformation results then from a compromise between landmark pairing and transformation regularity. Regardless, the proposed method generates a plausible 3D mesh of a baboon tongue from the 3D mesh of a living human tongue.

The second paragraph in the discussion section has been modified to clarify this aspect.

3. Could authors consider showing an image showing the segmented tongue of the human subject and the baboon? Seems rather difficult to see the geometry of the tongue of the baboon Figure 1(c), and is only the upper surface identifiable?

The segmentation illustrated in Fig. 2(a) actually corresponds to the outline of the FE tongue mesh depicted in Fig. 2(b, c). The caption of Fig. 2 was modified to clarify this aspect. We emphasize that the segmentation of tongue structures from CT information only is extremely challenging, since the boundaries between soft tissues are typically not visible. This work uses a FE tongue mesh built upon the fusion of multiple imaging modalities (CT, MRI, X-ray), as described in Sec. 2.1.2. For the Baboon, no segmentation nor FE meshing was possible, given that only CT data was available. For this reason, we used only a set of points from easily identifiable regions, as illustrated in Fig. 1(c, d).

4. Also, a related question, seems the tongue would deform easily, how would this compound the problem? E.g. necessary to have the human subj tongue at a similar position as a baboon (one would imagine it difficult to instruct baboon to do – forgive if this is too naïve question)

The reviewer raises a very good point. The registration result may indeed be affected by the initial position of the tongue in the images. However, it should be noted that this is not an issue for our application, since for a given position of bone structures a large number of tongue configurations exists. In other words, our focus is to predict a FE tongue mesh in a physically plausible tongue configuration considering only the bone structures, and not to predict specific, deformed tongue configurations.

References:

[Pearson1924] Pearson K., and Davin, A. G., “On the Biometric Constants of the Human Skull”, *Biometrika*, vol. 16, no. 3/4, p. 328, (1924)

[Iwasaki2019] Iwasaki, S.-I., Yoshimura, K., Shindo, J., and Kageyama, I., “Comparative morphology of the primate tongue,” *Annals of Anatomy*, vol. 223, pp. 19–31, (2019)

[Sotiras2013] Sotiras, A., Davatzikos, C., and Paragios, N., “Deformable Medical Image Registration: A Survey”, *IEEE Transactions on Medical Imaging*, vol. 32, no. 7, (2013)

[Rueckert2006] Rueckert, D., Aljabar, P., Heckemann, R. A., Hajnal, J. V., and Hammers, A., “Diffeomorphic Registration Using B-Splines”, in *Medical Image Computing and Computer-Assisted Intervention – MICCAI 2006*, Springer Berlin Heidelberg, (2006)

[Haghighi2018] Haghighi, B., Ellingwood, N. D., Yin, Y., Hoffman, E. A., and Lin, C.-L., “A GPU-based symmetric non-rigid image registration method in human lung”, *Medical and Biological Engineering and Computing*, vol. 56, no. 3, (2018)

[Calka2023] Calka, M. «*Modélisation biomécanique par éléments finis de la langue : évaluation, production de la parole et perspectives d’application à la chirurgie linguale assistée par ordinateur »*. *Diss. Université Grenoble-Alpes*, (2023)

[Buchaillard2007] Buchaillard, S., Brix, M., Perrier, P. & Payan, Y. (2007). Simulations of the consequences of tongue surgery on tongue mobility: Implications for speech production in post-surgery conditions. *International Journal of Medical Robotics and Computer Assisted Surgery,* Vol. 3(3), 252-261.

[Buchaillard2009] Buchaillard, S., Perrier, P., & Payan, Y. (2009). A biomechanical model of cardinal vowel production: muscle activations and the impact of gravity on tongue positioning. *Journal of the Acoustical Society of America*, 126(4), 2033-2051.
